# Time trends in episiotomy and severe perineal tears in Portugal: a nationwide register-based study

**DOI:** 10.1186/s12884-022-05314-6

**Published:** 2022-12-28

**Authors:** Cristina Teixeira, Elsa Lorthe, Henrique Barros

**Affiliations:** 1 EPIUnit - Instituto de Saúde Pública, Universidade Do Porto, Rua das Taipas nº135, 4050-600 Porto, Portugal; 2 Instituto Politécnico de Bragança, Bragança, Campus de Santa Apolónia, 5300-253 Bragança, Portugal; 3 Departamento de Ciências de Saúde Pública e Forenses e Educação Médica, Faculdade de Medicina da Universidade Do Porto, Alameda Prof. Hernâni Monteiro, 4200-319 Porto, Portugal

**Keywords:** Severe perineal tears, Episiotomy, Time-trends, Obstetric intervention, Women’s health

## Abstract

**Introduction:**

Rates of episiotomy and severe perineal tears (SPT) are indicators of the quality of obstetric care. Time-trends in the reported occurrence of episiotomy and SPT can contribute to understand both, changes in care and in the frequency of risk factors. Therefore, we aimed to estimate time trends in the frequency of SPT in Portugal and its relationship with episiotomy.

**Methods:**

We conducted a nationwide register-based study using data from the national inpatient database of all Portuguese public hospitals between 2000 and 2015. Time-trend analysis using joinpoint regression models was performed to identify trends (joinpoints) and compare time changes in the prevalence of SPT and risk factors expressed as annual percentage changes (APC) with 95% Confidence Intervals (95% CI). Poisson regression models were fitted to estimate whether time-trends in SPT rates were explained by changes in risk factors and to assess the association between episiotomy and SPT. Adjusted relative risk (aRR) and their respective 95% CI were obtained.

**Results:**

From 908,999 singleton vaginal deliveries, 20.6% were instrumental deliveries, 76.7% with episiotomy and 0.56% were complicated by SPT. Among women with non-instrumental deliveries and no episiotomy SPT decreased from 2009 onwards (1.3% to 0.7%), whereas SPT kept increasing in women with episiotomy for both non-instrumental (0.1% in 2000 to 0.4% in 2015) and instrumental deliveries (0.7% in 2005 to 2.3% in 2015). Time-trends in potential risk factors did not explain the observed increase in SPT. Episiotomy was associated with a decrease in SPT with adjusted RR varying between 2000 and 2015 from 0.18 (95%CI:0.13–0.25) to 0.59 (95%CI:0.44–0.79) for non-instrumental deliveries and from 0.45 (95%CI:0.25–0.81) to 0.50 (95%CI:0.40–0.72) for instrumental deliveries.

**Conclusions:**

Our findings suggest that episiotomy rate could safely further decrease as the main factor driving SPT rates seems to be an increase in awareness and reporting of SPT particularly among women who underwent an episiotomy.

## Introduction

Third- and fourth-degree perineal tears are severe complications of vaginal delivery [1] that are associated with increased risk of adverse outcomes including fecal incontinence [2, 3], urinary incontinence [4] and sexual dysfunction [5], with a negative impact on quality of life.

Episiotomy was introduced in clinical practice to ease delivery and to prevent perineal tears. However, its benefits remain unproven while its risks are well-known (haemorrhage, pain, dyspareunia) [6]. A systematic review of randomized controlled trials showed that selective episiotomy during non-instrumental vaginal delivery resulted in a reduction of severe perineal trauma compared with routine episiotomy [7]. These findings are likely to modify clinical practices in order to reduce the use of episiotomy, at least in non-instrumental deliveries [8].

In the 2010 Euro-Peristat data (20 countries), SPT rates ranged from 0.1% in Romania to 4.9% in Iceland, and episiotomy rates from 3.7% in Denmark to 75.0% in Cyprus. with a negative correlation between the rates of episiotomy and SPT by country. However, when considering rate differences for episiotomy and for SPT between 2004 and 2010, no correlation was observed between relative changes of SPT and episiotomy over time [9]. This observation raises the question of whether a decrease in episiotomy rates could have an impact on the variation of SPT rates, or whether the variation in SPT rates is primarily due to other factors rather than the use of episiotomy. Portugal is a country displaying a decreasing but still high rate of episiotomy (66.9% with non-instrumental deliveries and 94.4% with instrumental deliveries in 2010) and increasing SPT rates [8]. However, there is no epidemiological assessment of how changes in rates of SPT observed over time in Portugal are related to modifications in the prevalence of episiotomy and other determinants of SPT.

Using a national comprehensive database, which collects information on all admissions for delivery in public hospitals, we aimed to estimate time trends in the frequency of both SPT and episiotomy in Portugal, and to assess the relationship between episiotomy and SPT, taking into account the variation overtime in the frequency of known risk factors for SPT.

## Methods

### Context

In Portugal, where nearly all deliveries occur within hospitals, the National Health System provides antenatal, obstetric and neonatal care funded by public resources free of charge for all childbearing women and their babies. Although there is also a market supply of private health care services, public hospitals in Portugal cover a large majority of all deliveries (94% in 2000 and 85% in 2015) [10].

Labour and delivery are managed predominantly by doctors, including resident and attending obstetricians, but nurses specialised in maternal health, obstetrics and gynaecology, who support birthing in hospitals, play an increasing role in Portuguese maternity units [11]. These nurses are qualified to assist low-risk vaginal deliveries and were responsible for almost 40% of these deliveries in public hospitals in 2014 [12].

### Database and data collection

For the present study, we used data on all delivery related-admissions to Portuguese public hospitals between 2000 and 2015, obtained from the National Inpatient Database which is provided by the Portuguese Central Administration of the Health System. This database contains up to 20 diagnosis fields and up to 20 procedure fields for each discharge, coded by medical staff according to the Diagnosis Related Groups (DRG) [13] and the 9^th^ International Classification of disease (ICD-9) [14].

During the study period, there were 1,329,064 delivery related-discharges (DRG codes: 370–375, 540–542, 560 and 650–652). After excluding caesarean deliveries (*n* = 405,416), singleton pregnancies with fetal death (*n* = 5,560), multiple pregnancies (*n* = 5,974) and deliveries with no information about the number of babies or vital status of the child at birth (*n* = 3,115), we included in the present analysis 908,999 vaginal deliveries of singleton live born singletons.

The main outcome was SPT, identified by ICD-9 diagnosis codes 664.2x, 664.3x (third- and fourth-degree perineal tears), and 664.6x (anal sphincter tear not associated with third-degree perineal tears). Procedure codes 73.6 (episiotomy), 72.1, 72.21, 72.31 (low, mid and high forceps operation with episiotomy) and 72.71 (vacuum extraction with episiotomy) were used to identify women who underwent an episiotomy and/or an instrumental delivery.

The following risk factors for SPT [15, 16] were considered: mode of delivery dichotomized into instrumental, (including vacuum and forceps deliveries, (procedure codes 72.0 × to 72.4 × and 72.6 × to 72.9 × and diagnosis codes 669.5x), and non-instrumental vaginal delivery (all other vaginal deliveries), primiparous women ≥ 35 years old (diagnosis codes 659.5x), previous caesarean-section (diagnosis codes 654.2x), induced labor (procedure codes: 73.0 × to 73.4x, 75.0 and 96.49 and diagnosis codes: 658.3x, 659.0 × and 659.1x), epidural analgesia (procedure codes: 39.x), anomalous presentation or malposition of fetus (diagnosis codes: 652.0 × to 652.9x, 669.6 × and procedure codes: 72.5x), large baby for gestational age (diagnosis code: 656.6x), and materno-fetal disproportion (diagnosis codes: 653.0 × to 653.9x), dystocia/obstructed labor (diagnosis codes: 660.0 × to 660.4 × and 660.6 × to 660.9x) and long labor (diagnosis codes: 662.0 × to 662.3x).

### Statistical analyses

We calculated the prevalence of SPT (per 1,000 deliveries) and potential risk factors, including episiotomy (per 100 deliveries) by year between 2000 and 2015, among women with spontaneous deliveries, and then among women with instrumental deliveries. Analyses were also stratified by episiotomy use.

We evaluated time-trends for SPT and risk factors by using joinpoint regression models. Joinpoints are estimated iteratively; a joinpoint is a knot at which a significant change in the time-trend occurs [17]. The estimated annual percent change (APC) and their respective 95% Confidence Interval (95% CI) were obtained by fitting a regression line to the natural logarithm of the rates using calendar year as a regressor variable [17, 18]. The segment between two consecutive joinpoints corresponds to a trend characterized by a specific APC. Regression analysis was performed using the Joinpoint Regression Program, V.4.3.1.0 [18].

Then, we used multivariate Poisson regression models, adjusted for the potential risk factors defined previously, to investigate whether variation in risk factors could explain time-trends in rates. SPT was modeled as a function of time (calendar year), stratified by mode of delivery and episiotomy use. Finally, the association between episiotomy and SPT was assessed for each calendar year within each group according to the mode of delivery. Adjusted relative risk (RR) and 95%CI were obtained.

This statistical analysis was performed with SPSS software package version 25.0 and the level of significance was set at *p* < 0.05.

### Ethics approval

The study was approved on 27 June 2019 by the Ethics Committee of the Instituto de Saúde Pública da Universidade do Porto, Porto, Portugal, where the research was conducted (CE 19,121). The study was conducted on already available data on delivery-related discharges obtained from the National Inpatient Database provided by the Portuguese Central Administration of National Health System (Administração Central dos Serviços de Saúde, Portugal–ACSS).

## Results

From all 908,999 vaginal deliveries of singleton live infants during the study period, 186,931 (20.6%) were instrumental deliveries; 697,508 (76.7%) women underwent an episiotomy; and 5,129 deliveries (5.6 per 1000) were complicated by SPT.

**Fig. 1:**
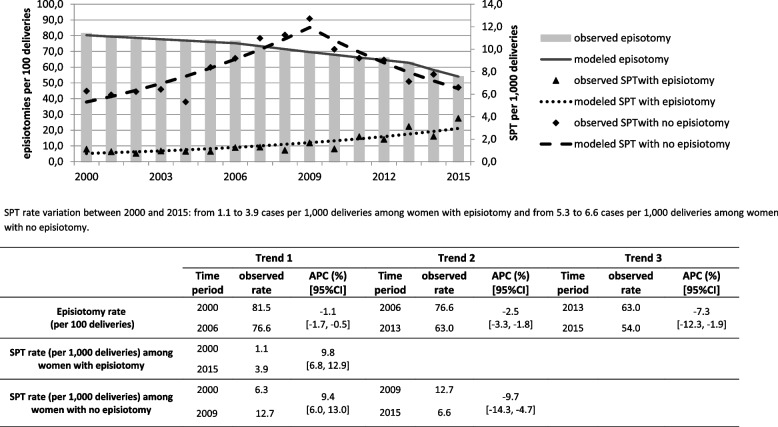
Time-trends in rates of episiotomy and SPT among 722 068 women having a non-instrumental delivery. SPT: Severe Perineal Tears. APC: annual percent change. Trend is the segment between two consecutive joinpoints (calendar years) characterized by a specific APC

**Fig. 2:**
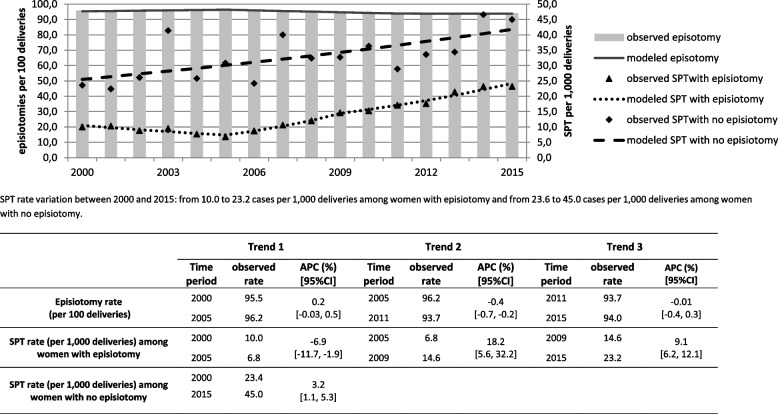
Time-trends in rates of episiotomy and rates of SPT among 186 931 women with instrumental delivery. SPT: Severe Perineal Tears. Trend is the segment between two consecutive joinpoints (calendar years) characterized by a specific APC APC: annual percent change

Figures 1 and 2 display temporal variation in rates of SPT and episiotomy among women with non-instrumental and with instrumental delivery, respectively. Rates of SPT were higher among women with no episiotomy regardless of the mode of delivery. The variation in SPT rates over time was differed according to the mode of delivery and the episiotomy use. Among women with a non-instrumental delivery and an episiotomy, SPT significantly increased over the whole study period from 1.1 to 3.9 per 1000 corresponding to an APC of 9.8% (95%CI: 6.8, 12.9). Women with non-instrumental deliveries and no episiotomy experienced a significant increase in SPT from 6.3 to 12.7 per 1,000 up to 2009 (APC = 9.4%; 95%CI: 6.0, 13.0) followed by a significant downward trend thereafter (APC = -9.7%; 95%CI: -14.3, -4.7) to reach 6.6 per 1000 in 2015 (Fig. 1). The incidence of episiotomy among women with non-instrumental delivery decreased from 81.5% in 2000 to 54.0% in 2015 and joinpoint analysis identified three different time-periods corresponding to 2000–2006 (APC = -1.1%; 95%CI: -1.7, -0.5), 2006–2013 (APC = -2.5%; 95%CI: -3.3, -1.8) and 2013–2015 (APC = -7.3%; 95%CI: -12.3, -1.9) as shown in Fig. 1.

Among women having an instrumental delivery and an episiotomy, SPT significantly decreased from 10.0 to 6.8 per 1000 up to 2005 corresponding to an APC of -6.9% (95%CI: -11.7, -1.9) and then significantly increased by 18.2% per year (95%CI: 5.6, 32.2) between 2005 and 2009 and 9.1% (95%CI: 6.2, 12.1) thereafter, to reach 23.2 per 1000 in 2015. Among women with an instrumental delivery but no episiotomy, representing less than 7% of women with instrumental deliveries, SPT rate significantly increased from 23.6 in 2000 to 45.0 per 1000 in 2015 (APC = 3.2%; 95%CI: 1.1, 5.3). The incidence of episiotomy among women having an instrumental delivery was high and almost stable (varying from 95.6% in 2000 to 94.0% in 2015) with three distinct time-periods but with a significant reduction only from 2005 to 2011 (APC = -0.4%; 95%CI: -0.7, -0.2) as shown in Fig. 2.

**Fig. 3:**
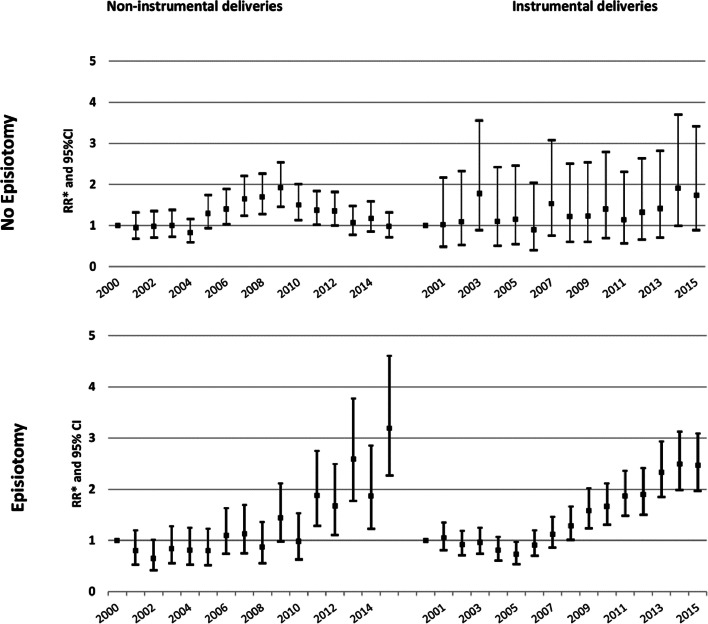
Adjusted relative risk for the association between calendar-year and SPT; 2000 as reference. * Adjusted for elderly primiparous, previous caesarean-section, induced labour, anomalous presentation or malposition of fetus, dystocia/disproportion/anomalous labour, baby large for gestational age and epidural anesthesia

Figure 3 displays adjusted RR and respective 95%CI for the association between calendar-year and SPT. Among women with no episiotomy no changes were found for instrumental deliveries, while for non-instrumental deliveries SPT almost doubled in 2009 in comparison with 2000 (RR = 1.92; 95%CI: 1.45–2.54), but reverted thereafter so that in 2015 was similar to 2000 (RR = 0.98; 95%CI: 0.72–1.32). Among women who underwent an episiotomy, the risk of SPT in 2015 was threefold higher compared to 2000 for non-instrumental deliveries (RR = 3.19; 95%CI: 2.27–4.60) and twofold higher for instrumental deliveries (RR = 2.47; 95%CI: 1.97–3.09).

As presented in Tables 1 and 2, during the study period, we observed marked changes in the clinical characteristics of women. In both groups by mode of delivery, there were significant increases in the frequency of primiparous women aged 35 or older over the whole time period (from 0.2% to 1.6% among women having non-instrumental and from 0.7% to 4.1% among those having instrumental delivery), the frequency of induced labor from 2006 onwards, (from 15.5% to 24.7% and from 20.8% to 31.2% among women having non-instrumental and instrumental delivery, respectively), while significant decreases were observed in the proportion of women delivering babies large for gestational age from 2004 (2.1% to 1.3% for non-instrumental vaginal delivery) or 2005 (2.8% to 1.6% for instrumental vaginal delivery) onwards. There were significant increases in the use of epidural analgesia over the whole time period among women with non-instrumental vaginal delivery (from 7.7% to 73.9%) and up to 2011 (from 18.3% to 86.7%) among women with instrumental vaginal delivery (Tables 1 and 2). Among women with a non-instrumental delivery, there were upward trends in the frequency of previous caesarean section from 2000 to 2006 (1.0% to 1.9%) and from 2009 (1.7% to 4.1%) onwards (Table 1). Among women having an instrumental delivery, there were significant increases in the frequency of previous caesarean section over the whole time period (3.4% to 8.5%) and dystocia (33.4% to 50.4%) from 2007 onwards (Table 2).

**Table 1:** Annual percent change (APC) in the prevalence of clinical characteristics among 722 068 women having a non-instrumental vaginal delivery in Portuguese public hospitals, 2000 – 2015

Clinical Characteristics	Trend 1			Trend 2			Trend 3			Trend 4		
	**Time period**	**prevalence %**	**APC (%) [95%CI]**	**Time period**	**prevalence %**	**APC (%) [95%CI]**	**Time period**	**prevalence %**	**APC (%) [95%CI]**	**Time period**	**prevalence %**	**APC (%) [95%CI]**
**Primiparous women ≥ 35 years old**	2000	0.2	12.0 [10.0; 14.0]									
	2015	1.6										
**Previous caesarean section**	2000	1.0	10.7 [5.7; 16.1]	2006	1.9	-2.3 [-23.7; 25.2]	2009	1.7	15.1 [11.0; 19.3]			
	2006	1.9		2009	1.7		2015	4.1				
**Induced labour**	2000	14.8	9.1 [-4.2; 24.3]	2002	17.5	-3.0 [-9.5; 3.8]	2006	15.5	5.0 [3.7; 6.3]			
	2002	17.5		2006	15.5		2015	24.7				
**Anomalous presentation or malposition of fetus**	2000	0.7	-6.7 [-10.1; -3.3]	2010	0.4	12.0 [-1.7; 27.6]						
	2010	0.4		2015	0.6							
**Disproportion/obstructed labor/long labor**	2000	0.9	-5.3 [-16.3; 7.2]	2004	0.7	11.5 [7.1; 16.1]	2013	2.1	-14.7 [-40.8; 22.9]			
	2004	0.7		2013	2.1		2015	1.5				
**Large for gestational age newborn**	2000	1.2	19.9 [6.4; 33.0]	2004	2.1	-6.7 [-9.2; -4.0]						
	2004	2.1		2015	1.3							
**Epidural analgesia**	2000	7.7	30.7	2005	29.3	18.0	2008	48.5	11.2	2011	65.8	2.6
	2005	29.3	[28.4; 33.0]	2008	48.5	[12.2; 24.0]	2011	65.8	[6.6; 16.0]	2015	73.9	[1.3; 4.0]

Trend is the segment between two consecutive joinpoints (calendar years) characterized by a specific APC

**Table 2:** Annual Percent change (APC) in the prevalence of clinical characteristics among 186 931 women having an instrumental delivery in Portuguese public hospitals, 2000 – 2015

Clinical Characteristics	Trend 1			Trend 2			Trend 3			Trend 4		
	**Time period**	**prevalence %**	**APC (%) [95%CI]**	**Time period**	**prevalence %**	**APC (%) [95%CI]**	**Time period**	**prevalence %**	**APC (%) [95%CI]**	**Time period**	**prevalence %**	**APC (%) [95%CI]**
**Primiparous women ≥ 35 years old**	2000	0.7	13.1 [11.4; 14.8]									
	2015	4.1										
**Previous caesarean section**	2000	3.4	2.5 [0.4; 4.7]	2009	4.4	11.3 [8.0; 14.7]						
	2009	4.4		2015	8.5							
**Induced labour**	2000	20.0	6.0 [-0.1; 12.6]	2003	24.1	-4.4 [-15.3; 7.8]	2006	20.8	4.1 [3.1; 5.2]			
	2003	24.1		2006	20.8		2015	31.2				
**Anomalous presentation or malposition of fetus**	2000	2.1	-6.6 [-8.5; -4.6]	2010	1.1	4.5 [-2.2; 11.7]						
	2010	1.1		2015	1.3							
**Disproportion/obstructed /long labor**	2000	36.5	-1.1 [-3.5; 0.4]	2007	33.4	7.6 [4.1; 11.2]	2012	48.7	1.4 [-3.3; 6.2]			
	2007	33.4		2012	48.7		2015	50.7				
**Large for gestational age newborn**	2000	2.1	4.9 [-2.6; 13.0]	2005	2.8	-6.5 [-9.2; -3.8]						
	2005	2.8		2015	1.6							
**Epidural analgesia**	2000	18.3	22.0	2004	41.1	12.2	2008	64.3	9.7	2011	84.2	0.3
	2004	41.1	[20.5; 23.5]	2008	64.3	[10.7; 13.8]	2011	84.2	[7.2; 12.2]	2015	86.7	[-0.3; 1.0]

Trend is the segment between two consecutive joinpoints (calendar years) characterized by a specific APC

Table 3 presents adjusted RR for the association between episiotomy and SPT according to the mode of delivery and by calendar year. Episiotomy was associated with a decrease in SPT whichever the calendar year, with RR varying between 2000 and 2015 from 0.18 (95%CI: 0.13–0.25) to 0.59 (95%CI: 0.44–0.79) for non-instrumental deliveries and from 0.45 (95%CI: 0.25–0.81) and 0.50 (95%CI: 0.40–0.72) for instrumental deliveries.

**Table 3:** Adjusted Relative Risk for the association between episiotomy and SPT by calendar year

	**Adjusted RR**^**a**^** (95%CI)**	
	**Non-instrumental delivery**	**Instrumental delivery**
**2000**	0.18 (0.13 – 0.25)	0.45 (0.25 – 0.81)
**2001**	0.15 (0.10 – 0.22)	0.43 (0.24 – 0.77)
**2002**	0.12 (0.08 – 0.18)	0.33 (0.19 – 0.58)
**2003**	0.15 (0.10 – 0.22)	0.23 (0.14 – 0.38)
**2004**	0.17 (0.11 – 0.26)	0.28 (0.15 – 0.53)
**2005**	0.11 (0.07 – 0.16)	0.26 (0.14 – 0.49)
**2006**	0.14 (0.10 – 0.20)	0.34 (0.18 – 0.64)
**2007**	0.12 (0.09 – 0.17)	0.27 (0.17 – 0.42)
**2008**	0.09 (0.06 – 0.13)	0.39 (0.24 – 0.64)
**2009**	0.13 (0.10 – 0.18)	0.45 (0.28 – 0.72)
**2010**	0.11 (0.08 – 0.17)	0.44 (0.29 – 0.68)
**2011**	0.24 (0.18 – 0.32)	0.61 (0.40 – 0.94)
**2012**	0.22 (0.16 – 0.31)	0.52 (0.34 – 0.80)
**2013**	0.43 (0.31 – 0.59)	0.57 (0.37 – 0.88)
**2014**	0.29 (0.20 – 0.41)	0.50 (0.35 – 0.71)
**2015**	0.59 (0.44 – 0.79)	0.50 (0.40 – 0.72)

^a^adjusted for primiparous women aged 35 or older, previous caesarean-section, induced labour, anomalous presentation or malposition of fetus, dystocia/disproportion/anomalous labor, baby large for gestational age and epidural anesthesia

## Discussion

In Portuguese public hospitals, there was a decrease in SPT among women with non-instrumental deliveries and no episiotomy (from 2009 onwards), whereas SPT kept increasing in women with episiotomy. Time-trends in potential risk factor did not s explain the observed increase in SPT. The overall frequency of SPT remains higher among women without than with episiotomy.

Although the frequency of episiotomy in non-instrumental deliveries remains high in Portugal, we observed a significant decrease over time. Large differences in episiotomy rates have been reported across European countries, varying from 5% in Denmark to 73% in Portugal [19], which probably reflects opposite opinions regarding the routine use of episiotomy. However, a consistent shift toward a restrictive use of episiotomy has become evident around the world over the last decade [8], denoting increased adherence to evidence-based practices [7]. This change in clinical practices may explain the trends in episiotomy rates we observed. In Europe, the proportion of women reported to have SPT after vaginal delivery ranges from 0.5% to 5.0% [9] and such variation has been partially explained by differences in patient characteristics [20, 21], hospital-related factors [20, 22] and clinical practices including the rates of instrumental delivery [22, 23], and the use of episiotomy [20, 22–24]. Also, the variation in assessment and reporting of SPT is considered an important issue in explaining the differences in SPT rates between settings [21–24]. Indeed, a non-negligible proportion of women with at least one vaginal delivery and no clinical diagnosis of SPT have an anal sphincter defect diagnosed by ultrasonography [3], indicating a potential underreport of SPT. Differences in the quality of such diagnosis could explain the variations in SPT rates across settings [21, 22, 24]. Likewise, increased awareness and training for detection of severe perineal tears over time plays a role in improving the diagnosis of perineal damages and leads to an increase in the reported SPT rate [21, 24]. As previously reported [21, 25–27], the improvement in the diagnosis of third- and fourth-degree perineal tears is an important contributor to the increase in SPT rate over time and it may explain the upward trend we observed in this study.

Our approach based on stratified time-trends analysis by the use of episiotomy allowed us to demonstrate that increases in SPT was evident among women with episiotomy, and according to our multivariate analyses, potential risk factors did not explain these upward time-trends. Instead, among women with no episiotomy, the upward trend in SPT rates reverted among women with non-instrumental delivery or appears explained by risk factors among those with an instrumental delivery. A study conducted in Finland also revealed an increase in SPT rates among women undergoing an episiotomy but a decrease or no change among women with no episiotomy [26]. However, in Finland the episiotomy rate is much lower than in Portugal (24% versus 70% in 2010) [9]. According to the results of the Finish study, the use of episiotomy became increasingly restricted to high risk women, which explain the upward trend in SPT [26]. In Portugal, the high episiotomy rate observed denote the routine use of the procedure likely based on the assumption that episiotomy has a protective effect against SPT. This assumption may have led, in the past, to healthcare providers paying less attention to the detection of SPT among women with episiotomy. Beyond the research published over the last decades on the potential harms of routine episiotomy [7], since 2001 it has been recommended that women having a vaginal delivery should have a digital rectal examination before suturing of the perineum [1]. The most likely consequence is the increased awareness in detecting and reporting of SPT particularly among women with episiotomy. Therefore, our results suggest that the rise in SPT is due to the increasing awareness of the recognition of perineal injuries, particularly in women with episiotomy.

Our findings revealed a protective effect of episiotomy against perineal damage for both non-instrumental and instrumental deliveries. Previous studies assessing the impact of episiotomy in the incidence of SPT yielded conflicting results. Episiotomy appears as a protective factor [16, 25, 27, 28], a risk factor [16, 20, 27] or a non-significant factor [20, 21] for the occurrence of SPT, as reported in different studies or in the same study across groups by women characteristics or by mode of delivery. Large heterogeneity in women characteristics [16, 20], criteria for selecting women for episiotomy [16], differences in episiotomy techniques [29] and the use of perineal protection techniques [30, 31] may explain the lack of consistent results, across settings and also over time, regarding the effect of episiotomy on SPT. However, the accuracy in diagnosing SPT seems be also a crucial issue when the assessment of the protective effect of the episiotomy is under discussion.

The main strength of this study is the analysis of a nationwide database, covering all delivery-related discharges from Portuguese public hospitals and corresponding to around 90% of deliveries in Portugal. Large databases provide appropriate sample sizes to study relatively rare outcomes, such as SPT. This database provided information on several diagnoses and procedures, which are potential risk factors for SPT. However, there are some limitations. A possible limitation in register-based data is the misclassification of diagnoses and the eventual change in accuracy of classification over time. However, non-random misclassification of diagnoses according to the use of episiotomy is unlikely. Another limitation is the lack of relevant information on ethnicity and parity (of the only variable available is primiparous woman ≥ 35 years old) which have been considered as risk factors for SPT [15]. We had no information on episiotomy techniques, the use of perineal protection procedures, or the type of healthcare professionals who provided obstetrical care. Because the type of episiotomy [29], as well as the use of manual perineal protection [15, 30, 31] have an effect on the risk SPT, the lack of information on these variables prevent us to assess if and how such factors changed during the time period under study and whether they could explain our results.

## Conclusions

In a country displaying a decreasing but still high rate of episiotomy, SPT rates showed a downward trend among women with non-instrumental deliveries and no episiotomy but they kept increasing in women with episiotomy. Our findings suggest that the rate of episiotomy could safely further decrease as the reason underlying the increase of SPT rate seems be a better ascertainment of SPT rather than the rate of episiotomy itself. Further research is needed to assess the accuracy of SPT diagnosis and to know the role of different episiotomy techniques in the SPT rate.

## Data Availability

The data that support the findings of this study were provided by the Portuguese Central Administration of National Health System (Administração Central dos Serviços de Saúde, Portugal–ACSS) from the National Inpatient Database. Data are not publicly available due to data protection issues. Restrictions apply to the availability of these data, which were used under license for the current study, but with no permission for data sharing. All methods were carried out in accordance with relevant guidelines and regulations.

## Abbreviation

SPT: Severe perineal tears
